# Four New Species and One New Record of Stromboscerini (Coleoptera, Curculionidae, Dryophthorinae) from China †

**DOI:** 10.3390/insects16121211

**Published:** 2025-11-28

**Authors:** Heyu Lü, Runzhi Zhang

**Affiliations:** 1 Key Laboratory of Animal Biodiversity Conservation and Integrated Pest Management, Chinese Academy of Sciences, No. 1 Beichen West Road, Chaoyang District, Beijing 100101, China; lvheyu23@ioz.ac.cn; 2 College of Life Science, University of Chinese Academy of Sciences, Beijing 100049, China

**Keywords:** diversity, morphology, Stromboscerini, taxonomy, type specimen, weevils

## Abstract

This study reports one genus newly recorded from China and four new species of the weevil tribe Stromboscerini. The genus *Dexipeus* Pascoe, 1885, is recorded in China for the first time, with one new species described: *Dexipeus tengchongensis* sp. nov. Additionally, three new species of *Tasactes* Faust, 1894, are recognized and diagnosed: *T. biunciatus* sp. nov., *T. liangi* sp. nov., and *T. sulcatus* sp. nov. For all new species, detailed morphological descriptions, diagnostic illustrations, and habitus pictures are provided. Two updated identification keys are presented. High-resolution pictures of the type specimens of the previously described Chinese species *Allaeotes niger* He, Zhang & Pelsue, 2003, and *Synommatoides scutellatus* He, Zhang & Pelsue, 2003, are also provided for future taxonomic research.

## Simple Summary

Small weevils of the tribe Stromboscerini inhabit forest floor litter in tropical and subtropical regions. Southern China is considered a diversity hotspot for this group, yet its true species richness has remained underestimated due to insufficient sampling. This study, based on valuable museum specimens, presents important new records and discoveries. The genus *Dexipeus* is newly recorded for China. Furthermore, four new species are diagnosed and described in the genera *Dexipeus* and *Tasactes*. Detailed habitus and morphological pictures are provided for all new taxa. This work increases the number of known Stromboscerini species in China to 15, substantially advancing our understanding of the group’s diversity in the region.

## 1. Introduction

The subfamily Dryophthorinae is widely accepted as a well-defined lineage within the family Curculionidae [1,2,3,4]. It comprises an estimated 1200 species classified into 153 extant genera and five tribes: Cryptodermatini, Dryophthorini, Stromboscerini, Orthognathini, and Rhynchophorini [4,5,6]. Among these, Stromboscerini represents a relatively small tribe containing 12 valid extant genera [7,8]. Its distribution is primarily restricted to a triangular area broadly delimited by Japan, Sri Lanka, and northern Australia [7]. Members of this tribe primarily inhabit the forest floor litter and are thought to be closely associated with Pinaceae (based on our specimen records, including *Orthosinus* from *Abies* forests). Recent phylogenetic studies have provided key evidence supporting the monophyly of Stromboscerini [2,9,10]. Taxonomically, works by Grebennikov [7,9,11,12] and Legalov [8,13,14,15,16,17,18,19,20] have substantially advanced the understanding of the tribe and established a solid research foundation.

Prior to this study, seven genera and 11 species of Stromboscerini were recorded from China, including *Allaeotes* Pascoe, 1885, *Dryophthoroides* Roelofs, 1879, *Nephius* Pascoe, 1885, *Orthosinus* Motschulsky, 1863, *Synommatoides* Morimoto, 1978, *Synommatus* Wollaston, 1873, and *Tasactes* Faust, 1894 [21,22]. The taxonomic study of Chinese Stromboscerini originates with the German entomologist Eduard Voss, who described *Orthosinus foveatus* Voss, 1953 and *Nephius salebrosus* (Voss, 1958) from Fujian [23,24]. Subsequently, a Chinese researcher systematically reviewed the group in an unpublished master’s thesis and formally described *Allaeotes niger* He, Zhang & Pelsue, 2003 (Jiangxi) and *Synommatoides scutellatus* He, Zhang & Pelsue, 2003 (Zhejiang) [25,26,27]. More recently, the present authors described four species of *Tasactes* from southwestern China [22].

Pascoe [28] established the genus *Dexipeus* Pascoe, 1885 for two species: *D. fumosus* from Java (designated as the type species by Lyal [29]) and *D. costatus* from Sumatra. Subsequently, Morimoto [30] reported *Dexipeus uenoi* from Okinawa, Japan, and Legalov [16] described *Dexipeus krasilnikovi* from the Philippines. To date, four species have been described in *Dexipeus*. The genus is diagnosed by the following combination of characteristics: eyes linear laterally, narrowly separated ventrally; antennal funicle 6-segmented, club obliquely truncate, tomentose apical surface flat; pronotum subcylindrical; elytra obovate, wider than pronotum; legs medium-sized, procoxae contiguous; femora unarmed; tibiae straight, bearing unci; tarsi slender, tarsomere 3 simple, not bilobed; claws free [28,30,31]. This genus had not been previously recorded from China. The genus *Tasactes* Faust, 1894 was erected by Faust [32] with *T. carinulatus* and *T. interruptus* (designated as the type species by Legalov [8]) from Myanmar. The genus is diagnosed by its transversely truncated antennal club with a conically projecting velvety apex, 6-segmented funicle, and distinctly ventrally separated eyes. It currently contains seven valid species distributed in Myanmar, Nepal, and China, four of which are recorded from China. Earlier studies indicated that numerous species of this genus in China remain undiscovered and undescribed [8].

In the present study, *Dexipeus* is recorded from China for the first time, with one new species described as *D. tengchongensis*. Additionally, three new species of *Tasactes* are discovered from Xizang and Yunnan, namely *T. biunciatus*, *T. huangi*, and *T. sulcatus*. This work provides diagnostic characters, detailed descriptions, and high-resolution pictures for all new species, along with updated identification keys. To facilitate future research, high-resolution pictures of the type specimens of *Allaeotes niger* He, Zhang & Pelsue, 2003 and *Synommatoides scutellatus* He, Zhang & Pelsue, 2003 are also provided.

## 2. Materials and Methods

The specimens examined in this study were discovered during a search of the museum’s collections. All specimens, including types, examined in this study, are deposited in the Institute of Zoology, Chinese Academy of Sciences, Beijing, China (IZCAS).

All morphological observations, measurements, and dissections were conducted using a Nikon SMZ 1500 stereomicroscope (Nikon, Minato City, Japan) with coaxial LED illumination. Specimens were imaged using a Canon EOS system (Canon, Tokyo, Japan), with detailed morphological features documented using a Canon 5D Mark II (Canon, Tokyo, Japan), and genitalia were documented using a Visionary Digital LK Lab System equipped with a Sony A7RM5 camera and a Mitutoyo 10× objective lens (Sony, Tokyo, Japan). Multi-focal image stacking was processed in Helicon Focus v. 7.5.4 Pro. All image plates were assembled and adjusted in Adobe Photoshop CC 2019. Labels written in Chinese are translated into English and cited verbatim.

Measurements follow Lü and Zhang [22], with the abbreviations: body length (Bl), rostrum length (Rl), rostrum width (Rw), pronotum length (Pl), pronotum width (Pw), elytra length (El), and elytra width (Ew = body width).

Terminology for general morphological structures primarily follows the online glossary of weevil characters proposed in the International Weevil Community Website (http://weevil.info/glossary-weevil-characters, accessed on 20 October 2025). Male genitalia terminology follows Oberprieler et al. [33], and terminology for certain leg structure follows Girón and Chamorro [34].

## 3. Results

### 3.1. Dexipeus Pascoe, 1885

Type species. *Dexipeus fumosus* Pascoe, subsequent designation by Lyal [29].
Diagnosis. see Introduction.
Distribution. China, Japan, Java, Indonesia, Philippines.

#### 3.1.1. Key to Species of the Genus *Dexipeus*

1. Elytral interstrial pubescence forming continuous pilose pustule…………………………………………………………………………………………………………………..2
-  Elytral interstrial pubescence interrupted, not forming pilose pustule……………………………………………………………………………………………………………..3
2. Forehead flat. Antennal club L/W ratio 1.7. Pronotum lacking median carina. Elytral sides subparallel. Body length 3.40–3.80 mm……………………...*D. krasilnikovi*
-  Forehead with small median fovea. Antennal club L/W ratio 2.0. Pronotum with fine median carina. Elytra widest at basal 1/3, sides not parallel. Body length 3.40–3.90 mm…………………………………………………………………………………………………………………………………………………………………………......*D. uenoi*
3. Body reddish brown. Rostrum shorter than pronotum, emarginate laterally at apical 1/3. Pronotal punctures small. Elytral interstriae with sparse pubescence. Body length 4.90 mm………………………………………………….……………………………………………………………………………………..*D. tengchongensis* sp. nov.
-  Body black. Rostrum not emarginate laterally. Pronotal punctures coarse. Elytral interstriae with dense pubescence. Body length 4.00 mm………………..………....4
4. Pronotal sides subparallel. Elytral punctures large, oblong-ovate.……………………………………………………………………………………………………... *D. costatus*
-  Pronotal sides weakly rounded. Elytral punctures very large, subquadrate…………………………………………………………………………………………... *D. fumosus*

#### 3.1.2. *Dexipeus tengchongensis* Lü & Zhang sp. nov.

**Figure 1:**
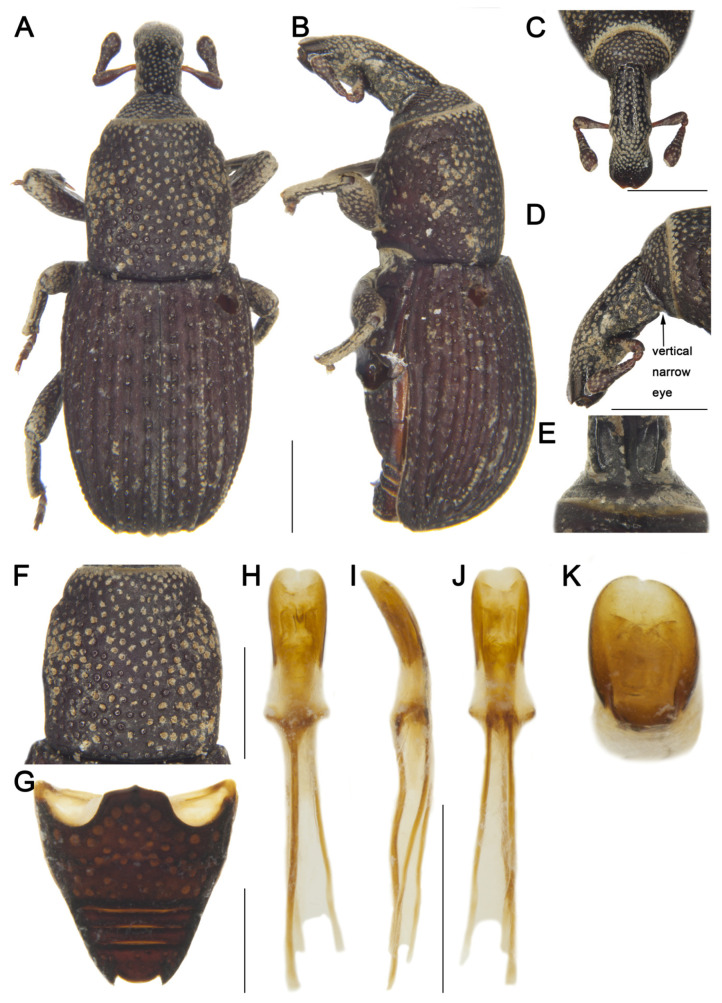
*Dexipeus tengchongensis* sp. nov., holotype male. (**A**) Dorsal habitus; (**B**) Lateral habitus; (**C**) Head, dorsal view; (**D**) Head, lateral view; (**E**) Eyes, ventral view; (**F**) Pronotum, dorsal view; (**G**) Ventrites, ventral view; (**H**) Penis, ventral view; (**I**) Penis, lateral view; (**J**) Penis, dorsal view; (**K**) Penis at apex, showing details of the pedon. Scale bars: 1 mm (**A**–**D**,**F**–**J**).

LSIDurn:lsid:zoobank.org:act:F38C1532-EEBB-485A-936C-E887C1802257
Figure 1
Material examined. Holotype: 1♀, China, Yunnan, Tengchong City, elev. 1920 m, 10/V/1979, collector unknown, IOZ(E)1507476.
**Comparative diagnosis.** *D. tengchongensis* sp. nov. is morphologically most similar to *D. costatus*, but differs in the following characters: (I) rostrum distinctly shorter than pronotum (Rl/Pl 0.73) (vs. longer than pronotum in *D. costatus*); (II) rostrum emarginate laterally at apical 1/3 (vs. lacking emargination in *D. costatus*); (III) pronotal punctures small, distance between punctures exceeding puncture diameter (vs. punctures large, distance less than puncture diameter in *D. costatus*); (IV) elytral interstriae with sparse pubescence, striae with small punctures and distance between punctures about 2.0 times the puncture diameter (vs. elytra with dense pubescence, striae with large punctures and distance subequal to puncture diameter in *D. costatus*; see Grebennikov [7]: figure 3A); (V) body reddish brown (vs. black in *D. costatus*); (VI) body larger (length 4.90 mm vs. 4.00 mm in *D. costatus*).
**Description. *Coloration*** (Figure 1A,B). Body reddish brown; head, mesosternum, and metasternum dark reddish brown; rostrum black.
***Head*** (Figure 1C–E). Forehead flat, narrower than rostrum, with deep depression between forehead and rostrum; eyes narrowly linear and vertical, length exceeding longest side of antennal club, widely separated ventrally; rostrum long (Rl/Rw 2.20), shorter than pronotum (Rl/Pl 0.73), emarginate laterally at apical 1/3 in dorsal view, nearly straight from base to apical 1/3 in lateral view, slightly curved from apical 1/3 to apex, base thick, from base to apical 1/5 with short pubescence and coarse punctures, apex smooth and shiny; antennae inserted at middle of rostrum; scape long (l/w 3.13), not reaching eyes, gradually broadening from base to apex, widest at apical 1/4; funicular segment 1 longer than wide, segment 2 funnel-shaped, segments 3–6 transverse; club subconical (l/w 1.50), apex obliquely truncate and densely pubescent.
***Pronotum*** (Figure 1F). Nearly cylindrical, longer than wide (Pl/Pw 1.10), widest at middle, apical 1/8 distinctly constricted, sides rounded; disc slightly convex in lateral view; densely covered with small punctures, distance between punctures exceeding puncture diameter; densely covered with short pubescence; postocular lobes absent.
***Scutellum*.** Small, invisible.
***Elytra*.** Obovate, longer than wide (El/Ew 1.45), widest at basal 1/4, apex nearly truncate, sides rounded; disc slightly convex in lateral view; interstriae distinctly convex, subequal in width, with 1–2 rows of short pubescence; striae as wide as interstriae, deep, punctures rounded, distance between punctures about 2.0 times puncture diameter.
***Abdomen*** (Figure 1G). Abdominal ventrites covered with coarse punctures, punctures on ventrites 3 and 4 sparser than on others, punctures at margins larger than those at middle; anterior margin of ventrite 2 slightly convex at middle, posterior margins of ventrites 2–4 rectilinear; ventrite 2 0.8 times length of ventrite 1, ventrite 3 as long as ventrite 4, ventrite 5 deeply emarginate, 2.5 times as wide as long.
***Legs.*** Densely covered with short pubescence; femora and tibiae with punctures; procoxae subconical, contiguous; mesocoxae narrowly separated; profemur more robust than mesofemur and metafemur, femora unarmed; tibiae bearing single long apical uncus; profemur 3.3 times as long as wide; protibia 5.0 times as long as wide; tarsi long, tarsomeres 1–3 obconical, ventrally with dense erect setae, onychium elongate; claws free, divergent.
***Male genitalia*** (Figure 1H–K). Pedon 0.3 times length of temones, gradually broadening from base to apical 1/3, then narrowed apically, curved in lateral view, sides rounded, base symmetrical; temones slender, slightly curved; manubrium of tegmen elongate, subequal to length of temones, slightly curved.
**Female:** unknown.
**Measurement (in mm).** Holotype. Bl: 4.90. Rl: 1.32, Rw: 0.60. Pl: 1.80, Pw: 1.63. El: 3.00, Ew: 2.10.
**Distribution.** Known only from the type locality in Yunnan, China.
**Etymology.** This species is named after its type locality, Tengchong City. Adjective, variable.

### 3.2. Tasactes Faust, 1894

Type species. *Tasactes interruptus* Faust, subsequent designation by Legalov [8].
Diagnosis. (modified from Lü and Zhang [22]): Body small; integument black to reddish brown, antennae and tarsomeres reddish-brown; head, rostrum, pronotum, abdominal ventrites densely covered with punctures; eyes linear or oval laterally, distinctly separated ventrally; antennal funicle 6-segmented, antennal club transversely truncated with conically projecting velvety apex; scutellum very small, usually subtriangular, sometimes not visible; elytra usually bearing pilose pustules, striae distinct; metepisternum entirely covered by elytra; legs usually covered with dense short pubescence; procoxae contiguous; femora unarmed; tibiae bearing 1–2 uncus; claws free; male genitalia usually without parameres, temones longer than pedon; lamina of female sternite 8 usually bifurcated; collum of spermatheca usually more developed than ramus.
Distribution. China, Myanmar, Nepal.

#### 3.2.1. Key to Species of the Genus *Tasactes* (Modified from Lü and Zhang [22])

1. Male protibia and mesotibia with only one uncus………………………………………………………………………………………………………………………………........2
-  Male protibia and mesotibia with two unci. Eyes barely visible in ventral view. Rostrum subequal to pronotum in length. Elytra widest at middle, interstriae with interrupted pilose pustules. Ventrite 5 with posterior margin convex. Body length 3.40–4.40 mm, width 1.31–1.80 mm………………………....... *T. biunciatus* sp. nov.
2. Elytra without interrupted pilose pustules………………………………………………………………………………………………………………………………………….....3
-  Elytra with interrupted pilose pustules……………………………………………………………………………………………………………………………………………........4
3. Rostrum shorter than pronotum. Pronotal sides rounded, lacking basal transverse de pression. Elytral interstriae subequal in width and height. Body length 3.40–4.40 mm, width 1.31–1.80 mm…………………………………………………………………………………..........................................................................................*T. angustus*
-  Rostrum longer than pronotum. Pronotal sides subparallel, with basal transverse depression. Elytral interstriae 1, 3 and 5 distinctly narrower than others. Body length 4.20 mm, width 1.80 mm……………………………………………………………………………...........................................................................................*T. carinulatus*
4. Pronotum punctate………………………………………..………………………………………………………………………………………………………………………..........5
-  Pronotum rugose-punctate. Body length 4.00–4.80 mm, width 1.81–2.20 mm……......................................................................................................................*T. interruptus*
5. Pronotum with weak postocular lobes ……………………………………………………………………………………………………………....................................................6
-  Pronotal postocular lobes absent ………………………………………………………………………………………………………………………………………………............8
6. Antennal scape L/W ratio 6.70. Pronotum with weak median carina, lacking median longitudinal pilose pustule. Body length 5.40–6.20 mm ……………...*T. dudkoi*
-  Antennal scape L/W ratio 4.70–4.75. Pronotum without median carina……………………………………………………………………………………………………….......7
7. Pronotum widest at apical 1/3, lacking median longitudinal pilose pustule; elytral pustules hemispherical in lateral view, densely distributed apically; styli width approximately 1/3 width of gonocoxite apices. Body length 5.22 mm, width 2.31 mm ....................................................................................................... *T. liangi* sp. nov.
-  Pronotum widest at middle, bearing median longitudinal pilose pustule; elytral pustules linear in lateral view, densely distributed; styli width approximately 1/4 width of gonocoxite apices. Body length 4.20–4.40 mm, width 1.91–1.98 mm ……………………………………………………………………………........…..……*T. pilosus*
8. Eyes linear in lateral view. Rostrum longer than pronotum. Antennal scape L/W ratio 5.18. Pronotum wider than long. Elytral pustules hemispherical in lateral view. Body length 4.20–4.40 mm, width 2.50–2.70 mm …………………………………………………………………………......................................................*T. baoxingensis*
-  Eyes oval in lateral view. Elytral pustules linear in lateral view………….…..……………………………………………………………………………………………………..9
9. Eyes barely visible in ventral view. Pronotum widest at middle, lacking sulci, spaces between punctures smooth. Scutellum visible. Elytral pubescence sparse. Pedon short, 0.3 times length of temones. Lamina of sternite 8 bifurcate at base, sides curved and depressed, apex with sparse setae. Styli width approximately 1/4 width of gonocoxite apices. Ramus of spermatheca not developed. Body length 3.65–3.89 mm, width 1.66–1.68 mm …………................................................*T. ocellatus*
-  Eyes distinctly visible in ventral view. Pronotum widest at apical 1/3, with sulci, spaces between punctures with pubescence. Scutellum invisible. Elytra with dense pubescence. Pedon long, 0.6 times length of temones. Lamina of sternite 8 truncatum, not bifurcate at base. Styli width approximately 1/3 width of gonocoxite apices. Ramus of spermatheca well developed. Body length 3.0–3.83 mm, width 1.30–1.60 mm ………………………………………….... *T. sulcatus* sp. nov.

#### 3.2.2. *Tasactes biunciatus* Lü & Zhang sp. nov.

**Figure 2:**
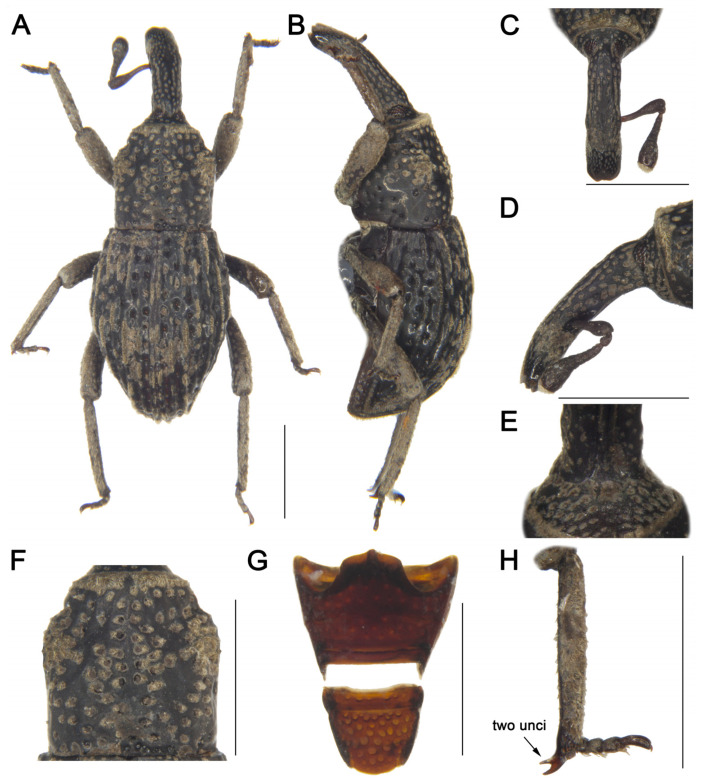
*Tasactes biunciatus* sp. nov., holotype male. (**A**) Dorsal habitus; (**B**) Lateral habitus; (**C**) Head, dorsal view; (**D**) Head, lateral view; (**E**) Eyes, ventral view; (**F**) Pronotum, dorsal view; (**G**) Ventrites, ventral view; (**H**) Protibia, showing details of the uncus. Scale bars: 1 mm (**A**–**D**,**F**–**H**).

**Figure 3:**
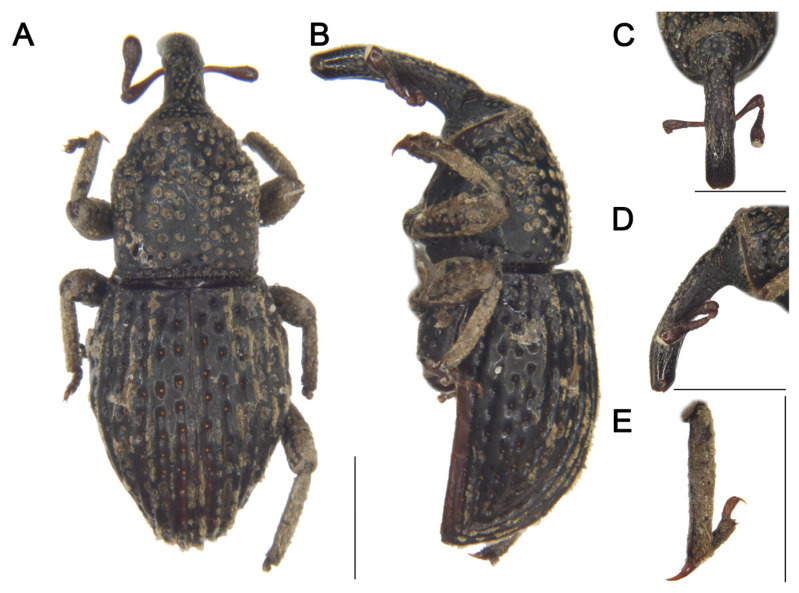
*Tasactes biunciatus* sp. nov., paratype female (IOZ(E)1507499). (**A**) Dorsal habitus; (**B**) Lateral habitus; (**C**) Head, dorsal view; (**D**) Head, lateral view; (**E**) Protibia, showing details of the uncus. Scale bars: 1 mm (**A**–**E**).

LSIDurn:lsid:zoobank.org:act:84CD1BB2-1A02-43D2-922E-D29341C557C8
Figure 2 and Figure 3
Material examined. Holotype: 1♂, China, Yunnan, 1956, collector unknown, IOZ(E)1507496. Paratypes: 2♂1♀, same data as holotype, IOZ(E)1507497–1507499.
**Comparative diagnosis.** *T. biunciatus* sp. nov. is a very peculiar species characterized by male protibia and mesotibia with two unci, which cannot be confused with any other species of the genus. Besides this character, its morphology is most similar to *T. ocellatus* Lü & Zhang, 2025, but differs in the following characters: (I) rostrum subequal to pronotum (Rl/Pl 1.00) (vs. rostrum shorter than pronotum (Rl/Pl 0.73) in *T. ocellatus*); (II) posterior margin of pronotum truncate (vs. convex at middle in *T. ocellatus*); (III) elytra widest at middle, pilose pustules not forming distinct pattern (vs. elytra widest at basal 1/4, pilose pustules forming indistinct V-shaped pattern in *T. ocellatus*); (IV) posterior margin of ventrite 5 convex (vs. deeply emarginate in *T. ocellatus*).
**Description. *Coloration*** (Figure 2A,B). Body entirely black; antennal scape, funicle, tarsomeres, and claws reddish brown.
***Head*** (Figure 2C–E). Forehead concave, narrower than rostrum; eyes oval, length shorter than longest side of antennal club, barely visible ventrally; rostrum long (Rl/Rw 3.42), subequal to pronotum in length (Rl/Pl 1.00), slightly curved in lateral view, base thick, from base to apical 1/5 with short pubescence and coarse punctures, apex smooth and shiny; antennae inserted slightly anterior to middle of rostrum; scape long (l/w 3.95), not reaching eyes, gradually broadening from base to apex, widest at apex; funicular segment 1 longer than wide, segment 2 funnel-shaped, segments 3–6 transverse; club subconical (l/w 1.65).
***Pronotum*** (Figure 2F). Nearly cylindrical, longer than wide (Pl/Pw 1.04), widest posterior to middle, apical 1/6 distinctly constricted, gradually narrowed from apical 1/4 to base; disc slightly convex in lateral view; densely covered with punctures, particularly concentrated on central disc and lateral areas; postocular lobes absent.
***Scutellum.*** Very small, elongate-oval.
***Elytra.*** Longer than wide (El/Ew 1.43), widest at middle, strongly narrowed from middle to apex, lacking constriction; disc slightly convex in lateral view; interstriae distinctly convex, subequal in width, with interrupted pilose pustules; striae deep, punctures rounded, with dense, short pubescence; distance between punctures slightly exceeding puncture diameter.
***Abdomen*** (Figure 2G). Abdominal ventrites covered with coarse punctures; distance between punctures exceeding puncture diameter on ventrites 1–4; punctures on ventrite 5 coarser than on others, distance between punctures smaller than puncture diameter; anterior margin of ventrite 2 slightly convex at middle, posterior margins of ventrites 3 and 4 rectilinear; ventrite 2 0.64 times length of ventrite 1, ventrite 4 slightly longer than ventrite 3, ventrite 5 convex, 1.5 times as wide as long.
***Legs.*** Densely covered with short pubescence; femora and tibiae with punctures; procoxae subconical, contiguous; mesocoxae narrowly separated; profemur more robust than mesofemur and metafemur, femora unarmed; protibiae and mesotibiae each bearing two adjacent apical unci (Figure 2H), metatibia with single long uncus; profemur 3.6 times as long as wide; protibia 6.9 times as long as wide; tarsi long, tarsomeres 1–3 obconical, ventrally with dense erect setae, onychium elongate; claws free, divergent.
***Female*** (Figure 3A–E). Body larger than that of male; rostrum slightly shorter than pronotum, from apical 1/3 to apex smooth and shiny, antennae inserted at middle of rostrum; interrupted pilose pustules on elytral interstriae 1 and 2 sparser than those of male; protibiae, mesotibiae and metatibiae each bearing only single long uncus.
**Measurement (in mm).** Holotype. Bl: 3.60. Rl: 1.20, Rw: 0.35. Pl: 1.20, Pw: 1.15. El: 2.20, Ew: 1.53. Male paratypes. (*n* = 2): Bl: 3.48–3.58 (3.53). Rl: 1.2, Rw: 0.35–0.38 (0.37). Pl: 1.2, Pw: 1.10–1.17 (1.14). El: 2.00–2.20 (2.10), Ew: 1.50–1.52 (1.51). Female paratype. Bl: 3.70. Rl: 1.32, Rw: 0.42. Pl: 1.35, Pw: 1.32. El: 2.25, Ew: 1.60.
**Distribution.** Known only from Yunnan, China.
**Etymology.** The species name is a Latin masculine adjective *biunciatus*, referring to the male tibiae bearing two unci compared to other species within the genus.

#### 3.2.3. *Tasactes liangi* Lü & Zhang sp. nov.

**Figure 4:**
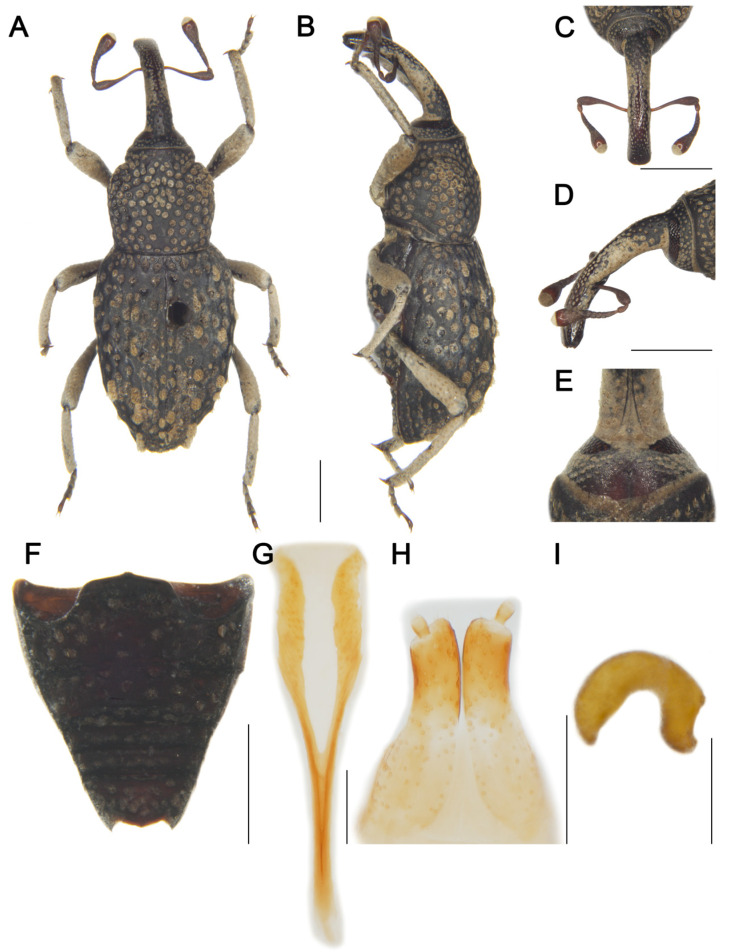
*Tasactes liangi* sp. nov., holotype female. (**A**) Dorsal habitus; (**B**) Lateral habitus; (**C**) Head, dorsal view; (**D**) Head, lateral view; (**E**) Eyes, ventral view; (**F**) Ventrites, ventral view; (**G**) Sternite 8; (**H**) Ovipositor; (**I**) Spermatheca. Scale bars: 1 mm (**A**–**D**,**F**); 0.25 mm (**G**–**I**).

LSIDurn:lsid:zoobank.org:act:13DF0B1A-059C-4361-BAD2-1D30840D485C
Figure 4
Material examined. Holotype: 1♀, China, Yunnan, Diqing Tibetan Autonomous Prefecture, Gongshan County, Cikai Town, Gazu Station, 27.7431° N, 98.6047° E, elev. 1500 m, 04/V/2002, Hongbin Liang & Weidong Ba leg. IOZ(E)1965838.
**Comparative diagnosis.***T. liangi* sp. nov. resembles *T. pilosus* Lü & Zhang, 2025 but differs in the following characters: (I) rostrum more curved (see Lü and Zhang [22]: figure 7D vs. Figure 4D); (II) pronotum widest at apical 1/3, lacking pilose pustule (vs. widest at middle, bearing median longitudinal pilose pustule in *T. pilosus*); (III) elytral pustules hemispherical in lateral view, sparsely distributed (vs. linear in lateral view, densely distributed in *T. pilosus*); (IV) styli width approximately 1/3 width of gonocoxite apices (vs. approximately 1/4 in *T. pilosus*).
**Description. *Coloration*** (Figure 4A,B). Body entirely black; antennal scape, funicle, tarsomeres, and claws reddish brown.
***Head*** (Figure 4C–E). Forehead flat, slightly narrower than rostrum base width; eyes oval, length exceeding longest side of antennal club, barely visible ventrally; rostrum elongate (Rl/Rw 3.52), longer than pronotum (Rl/Pl 1.43), evenly curved in lateral view, from base to apical 1/6 with short pubescence, apex with dense punctures; antennae inserted slightly anterior to middle of rostrum; scape long (l/w 4.70), not reaching eyes, gradually broadening from base to apex, apical 1/3 markedly widened; funicular segments 1 and 2 elongate, segment 2 funnel-shaped, longer than segments 3+4 combined; segments 3–6 transverse; club subconical (l/w 1.86).
***Pronotum.*** Wider than long (Pl/Pw 0.97), widest at apical 1/3, apical 1/7 distinctly constricted, gradually narrowed from apical 1/3 to base, sides rounded; disc convex in lateral view; densely covered with punctures, particularly concentrated on central disc and lateral areas; postocular lobes weak.
  ***Scutellum.*** Small, subtriangular.
***Elytra.*** Longer than wide (El/Ew 1.42), widest at basal 1/4, apical 1/6 distinctly constricted, sides rounded; disc nearly flat in lateral view; interstriae distinctly convex, subequal in width, basally with small punctures distinctly smaller than strial punctures, interstriae with interrupted oval pilose pustules, mainly distributed from apical 1/3 to apex; striae deep, punctures rounded, bearing dense, short pubescence; distance between punctures slightly exceeding puncture diameter.
***Abdomen*** (Figure 4F). Abdominal ventrites densely covered with coarse punctures; ventrite 2 with anterior margin slightly convex at middle, posterior margins of ventrites 2–4 rectilinear; ventrite 2 0.7 times length of ventrite 1, ventrite 3 slightly longer than ventrite 4, ventrite 5 deeply emarginate, 2.1 times as wide as long.
***Legs.*** Densely covered with short pubescence; femora and tibiae with punctures; procoxae subconical, contiguous; profemur more robust than mesofemur and metafemur, femora unarmed; profemur 3.9 times as long as wide; tibiae bearing single long uncus; protibia 5.9 times as long as wide; tarsi long, tarsomeres 1–3 obconical, ventrally with setae, onychium elongate; claws free, divergent.
***Sternite 8 and genitalia*** (Figure 4G–I). Sternite 8 with apodeme 0.9 times length of lamina; lamina bifurcate at base, sides curved, apex with sparse setae; gonocoxites cylindrical, apices with setae; styli short, cylindrical, width approximately 1/3 width of gonocoxite apices, apices with short setae; spermatheca with curved, apically rounded cornu; corpus robust; ramus and collum weakly developed.
**Male.** unknown.
**Measurement (in mm).** Holotype. Bl: 5.22. Rl: 2.00, Rw: 0.43. Pl: 1.75, Pw: 1.80. El: 3.28, Ew: 2.31.
**Distribution.** Known only from the type locality in Yunnan, China.
**Etymology.** This species is named after the professor of the Institute of Zoology, Chinese Academy of Sciences in China and also the collector of the type specimens, Dr. Hongbin Liang. The specific name is a noun in apposition.

#### 3.2.4. *Tasactes sulcatus* Lü & Zhang sp. nov.

**Figure 5:**
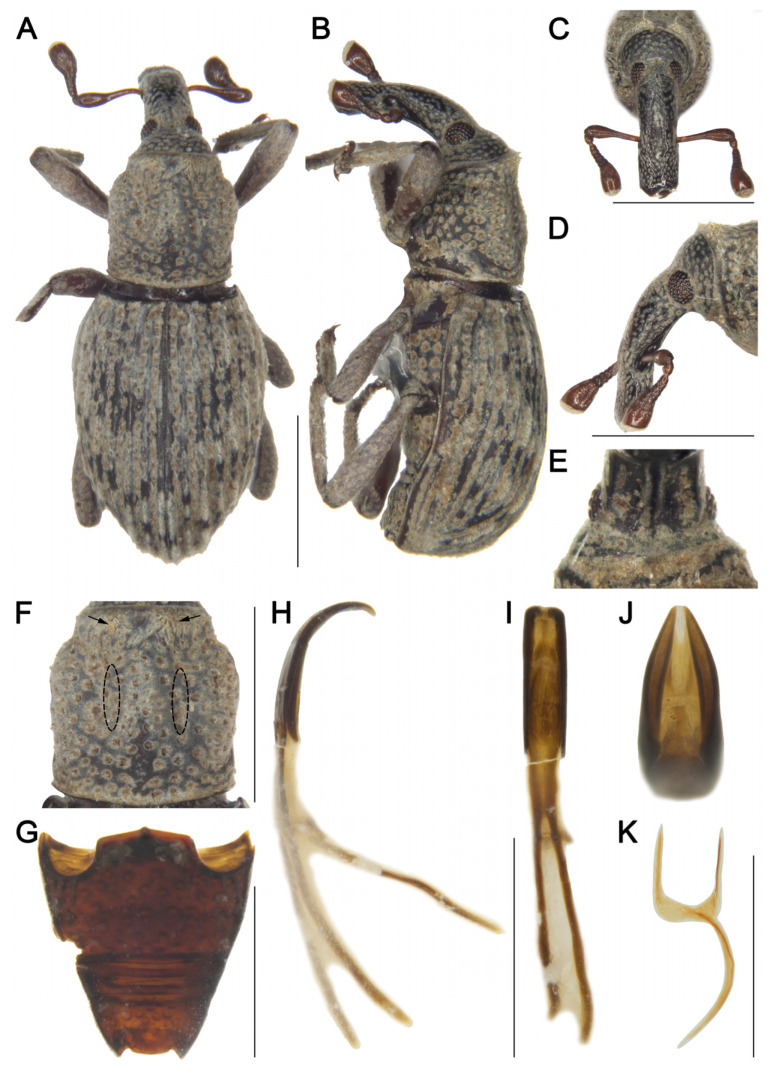
*Tasactes sulcatus* sp. nov., holotype male. (**A**) Dorsal habitus; (**B**) Lateral habitus; (**C**) Head, dorsal view; (**D**) Head, lateral view; (**E**) Eyes, ventral view; (**F**) Pronotum, dorsal view; (**G**) Ventrites, ventral view; (**H**) Penis, lateral view; (**I**) Penis, dorsal view; (**J**) Penis at apex, showing details of the pedon; (**K**) Spiculum gastrale. Scale bars: 1 mm (**A**–**I**,**K**).

**Figure 6:**
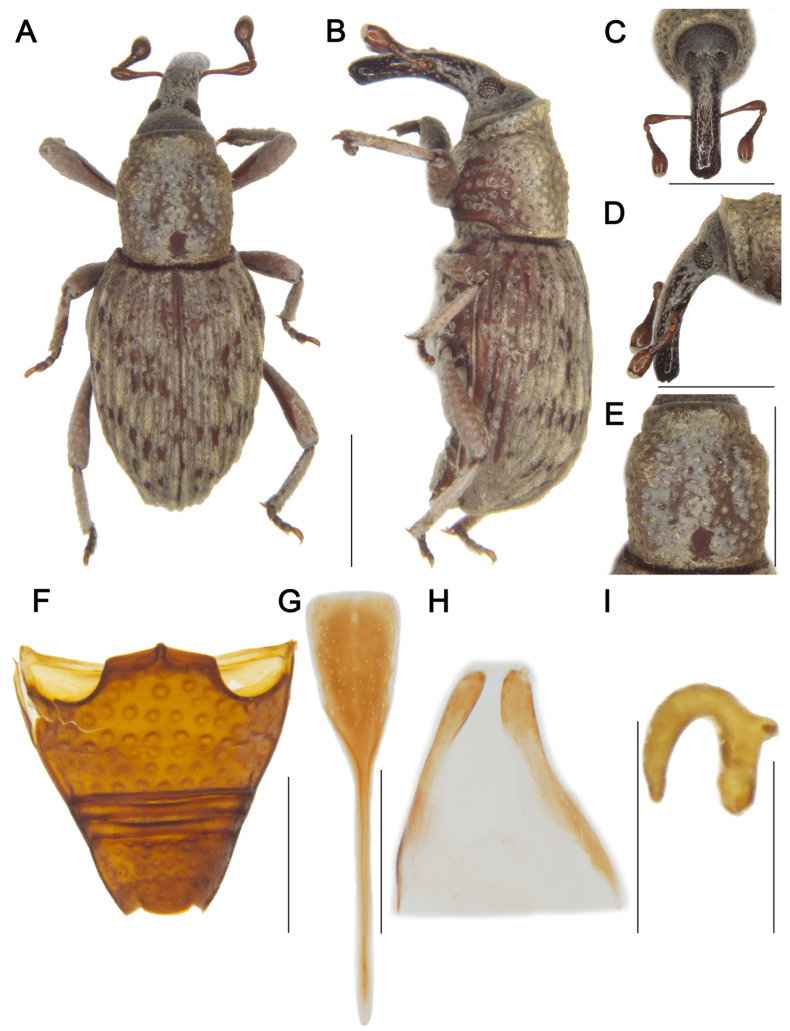
*Tasactes sulcatus* sp. nov., paratype female (IOZ(E)1965729). (**A**) Dorsal habitus; (**B**) Lateral habitus; (**C**) Head, dorsal view; (**D**) Head, lateral view; (**E**) Pronotum, dorsal view; (**F**) Ventrites, ventral view; (**G**) Sternite 8; (**H**) Ovipositor; (**I**) Spermatheca. Scale bars: 1 mm (**A**–**F**); 0.25 mm (**G**–**I**).

LSIDurn:lsid:zoobank.org:act:893E4281-1547-4C44-8A8A-C44700912287
Figure 5 and Figure 6
Material examined. Holotype: 1♂, China, Xizang, Linzhi City, Mêdog County, Beibeng Township, Doxong Pass, Lage hotel, 29.4675° N, 95.00232° E, elev. 3210 m, 07/VIII/2006, Hongbin Liang leg. IOZ(E)1965835. Paratypes: 12♀, same data as holotype, IOZ(E)1965728, 1965729, 1965827–1965834, 1965836, 1965837.
**Comparative diagnosis.***T. sulcatus* sp. nov. is most similar to *T. ocellatus*, which is also distributed in Mêdog County, but differs in the following characters: (I) eyes distinctly visible in ventral view (vs. barely visible in *T. ocellatus*); (II) pronotum widest at apical 1/3, spaces between punctures with pubescence, with sulci laterally to middle (vs. widest at middle, interpuncture spaces smooth, lacking sulci in *T. ocellatus*); (III) scutellum invisible (vs. visible in *T. ocellatus*); (IV) elytra with dense pubescence (vs. sparse in *T. ocellatus*); (V) pedon long, 0.6 times length of temones (vs. pedon short, 0.3 times length of temones in *T. ocellatus*); (VI) lamina of sternite 8 truncate, not bifurcate at base (vs. bifurcate at base, sides curved and depressed in *T. ocellatus*); (VII) styli width approximately 1/3 width of gonocoxite apices, ramus of spermatheca weakly developed (vs. approximately 1/4 width of gonocoxite apices, ramus well developed in *T. ocellatus*).
**Description. *Coloration*** (Figure 5A,B). Body entirely black; antennal scape, funicle, tarsomeres, and claws reddish brown.
***Head*** (Figure 5C–E). Forehead flat, slightly narrower than rostrum base width; eyes oval, shorter than longest side of antennal club, distinctly separated ventrally; rostrum elongate (Rl/Rw 3.52), shorter than pronotum (Rl/Pl 0.90), evenly curved in lateral view, from base to apical 1/3 with short pubescence, apex with dense punctures; antennae inserted slightly anterior to middle of rostrum; scape long (l/w 4.00), not reaching eyes, gradually broadening from base to apex, apical 1/3 markedly widened; funicular segments 1 and 2 elongate, segment 2 funnel-shaped, subequal in length to segments 3+4 combined; segments 3–6 transverse; club subconical (l/w 1.80).
***Pronotum*** (Figure 5F). Longer than wide (Pl/Pw 1.15), widest at apical 1/3, apical 1/6 distinctly constricted, gradually narrowed from apical 1/4 to base; disc slightly convex in lateral view, with dense and coarse punctures; distance between punctures subequal to puncture diameter, apical with two clusters of pilose pustules; with one broad oval sulcus on each side of the middle; postocular lobes absent.
***Scutellum.*** Small, invisible.
***Elytra.*** Longer than wide (El/Ew 1.46), widest at basal 1/4, apical 1/5 distinctly constricted, sides rounded; disc nearly flat in lateral view; interstriae and striae with dense short pubescence; interstriae distinctly convex, subequal in width, with interrupted pilose pustules; striae deep, punctures rounded, distance between punctures slightly less than puncture diameter.
***Abdomen*** (Figure 5G). Abdominal ventrites densely covered with coarse punctures; ventrite 2 with anterior margin slightly convex at middle, posterior margins of ventrites 2–4 rectilinear; ventrite 2 0.6 times length of ventrite 1, ventrites 3 and 4 subequal in length, ventrite 5 deeply emarginate, 2.0 times as wide as long.
***Legs.*** Densely covered with short pubescence; femora and tibiae with punctures; procoxae subconical, contiguous; profemur more robust than mesofemur and metafemur, femora unarmed; profemur 3.7 times as long as wide; tibiae bearing single long uncus; protibia 6.6 times as long as wide; tarsi long, tarsomeres 1–3 obconical, ventrally with setae, onychium elongate; claws free, divergent.
***Male genitalia*** (Figure 5H–K). Pedon 0.6 times length of temones, evenly curved in lateral view, sides subparallel, base symmetrical, apex distinctly narrowed; temones slender, slightly curved; manubrium of tegmen long, 0.9 times length of temones, slightly curved, subequal in width to temones; spiculum gastrale evenly curved, widest at middle; basal plate bifurcate, basal arms slender, opposed, apices acute.
***Female*** (Figure 6A–I). Body larger than in male, some individuals with lighter, reddish-brown body coloration; rostrum more longer than in male; antennae inserted at middle of rostrum; sternite 8 with apodeme 1.7 times length of lamina; lamina truncatum, not bifurcate at base, apex with short setae; gonocoxites cylindrical, apices with long setae; styli short, cylindrical, width approximately 1/3 width of gonocoxite apices, apices with short setae; spermatheca with curved, apically rounded cornu; corpus robust; ramus longer than collum.
**Measurement (in mm).** Holotype. Bl: 3.00. Rl: 0.88, Rw: 0.25. Pl: 0.98, Pw: 0.85. El: 1.90, Ew: 1.30. Female paratypes. (*n* = 12): Bl: 3.40–3.83 (3.65). Rl: 1.00–1.06 (1.02), Rw: 0.30–0.35 (0.31). Pl: 1.10–1.22 (1.15), Pw: 0.95–1.04 (1.04). El: 2.15–2.40 (2.26), Ew: 1.38–1.60 (1.42).
  **Distribution.** Known only from the type locality in Xizang, China.
**Etymology.** The species name is a Latin masculine adjective *sulcatus*, referring to the pronotum with broad oval sulci compared to other species within the genus.

### 3.3. Allaeotes Pascoe, 1885

Type species. *Allaeotes griseus* Pascoe, by original designation.
Diagnosis. See Morimoto [30,31] and He et al. [27].
Distribution. China, Cuba, Japan, Indonesia, Vietnam.

#### *Allaeotes niger* He, Zhang & Pelsue, 2003

**Figure 7:**
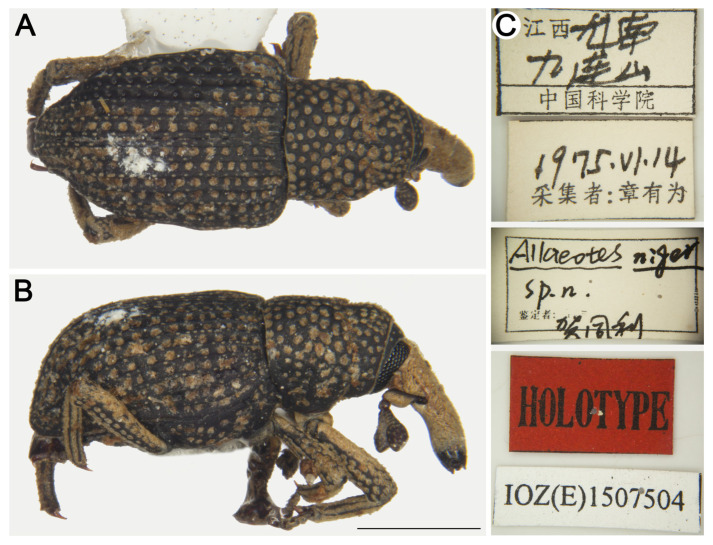
*Allaeotes niger*, holotype male. (**A**) Dorsal habitus; (**B**) Lateral habitus; (**C**) labels. Scale bars: 1 mm (**A**,**B**).

Figure 7
*Allaeotes niger* He et al. [27]: 127; Lyal [35]: 192 (cataloged); Alonso-Zarazaga et al. [21]: 244 (cataloged); Kojima & Fujisawa [36]: 10 (redescription); Grebennikov [12]: 10 (report).
Type material examined. Holotype: 1♂, China, Jiangxi, Longnan County, Jiulian Mountain, 14/VI/1975, Youwei Zhang leg. IOZ(E)1507504. Paratypes: 1♀, same data as holotype, IOZ(E)1507505; 1♀, Zhejiang, Hangzhou City, 12/VI/1954, collector unknown, IOZ(E)1507506; 1♀, Fujian, Fuan City, Shekou, 22/VI/1955, collector unknown, IOZ(E)1507507; 18♂12♀, Guangdong, Guangzhou City, 05/IV/1954, Keren Huang leg. IOZ(E)1507508–1507525 (some numbers correspond to multiple individuals).
  Additional material examined. 1♂, China, Hong Kong, collector unknown, IOZ(E)1965723; 3♂4♀, Guangxi, Laibin City, Jinxiuyaozu Autonomous County, Huawang Villa, elev. 600 m, 20/V/1999, Fusheng Huang leg. IOZ(E)1965724–1965727, 1965731–1965733.
**Remarks.** He et al. [27] distinguished this species from *A. griseus*. Based on examination of the type specimen and syntype images from Grebennikov [12], we document additional diagnostic characters differentiating *A. niger* from *A. griseus*: (I) head with distance between punctures smooth (vs. dense short pubescence in *A. griseus*); (II) rostrum slightly curved (Figure 7B) (vs. strongly curved at apical 1/3 in *A. griseus*; see Grebennikov [12]: figure 3H); (III) pronotum constricted at apical 1/3, with dense coarse punctures and and spaces between punctures smooth (vs. constricted at apical 1/5, with dense small punctures and spaces between punctures with short pubescence in *A. griseus*); (IV) elytral interstriae lacking interrupted pilose pustules (vs. interrupted pilose pustules in *A. griseus*). It is further distinguished from *A. sklodowskii* Legalov, 2021 by: (I) antennal club shorter and stouter (l/w 1.33 vs. l/w 1.50 in *A. sklodowskii*); (II) pronotal lacking median carina (vs. weak median carina in *A. sklodowskii*); (III) scutellum spindle-shaped (vs. triangular in *A. sklodowskii*); (IV) elytral interstriae lacking interrupted pilose pustules (vs. interrupted pilose pustules in *A. sklodowskii*).

### 3.4. Synommatoides Morimoto, 1978

Type species. *Synommatoides shirozui* Morimoto, by original designation.
Diagnosis. See Morimoto [30,31] and He et al. [26].
Distribution. China, Japan, Korea.

#### *Synommatoides scutellatus* He, Zhang & Pelsue, 2003

**Figure 8:**
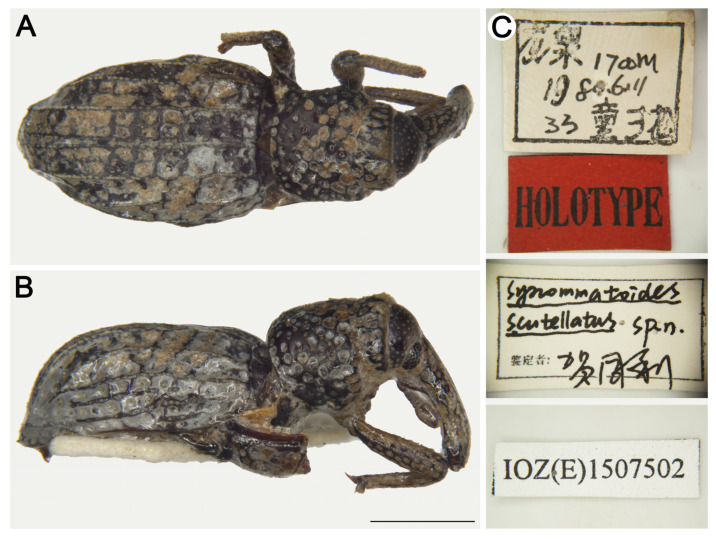
*Synommatoides scutellatus*, holotype female. (**A**) Dorsal habitus; (**B**) Lateral habitus; (**C**) labels. Scale bars: 1 mm (**A**,**B**).

Figure 8
*Synommatoides scutellatus* He et al. [26]: 123; Lyal [35]: 192 (cataloged); Alonso-Zarazaga et al. [21]: 244 (cataloged).
Type material examined. Holotype: 1♀, China, Zhejiang, Lishui City, Longquan County, Fengyang Mountain, elev. 1700 m, 11/VI/1980, Xuesong Tong leg. IOZ(E)1507502. Paratype: 1♂, same data as holotype, IOZ(E)1507503.
**Remarks.** He et al. [26] distinguished this species from *S. shirozui* by the following characters: scutellum present and tiny; apical margin of pronotum punctate with two pilose pustules; antennae stout and more compact. These characters are not diagnostic, as the apical margin of the pronotum in *S. shirozui* also bears two pilose pustules. Based on the present study of the type specimen of *S. scutellatus* and the holotype images of *S. shirozui* from Grebennikov [7], we identify the following reliable characters to distinguish the two species: (I) pronotum not rugose-punctate (vs. rugose-punctate in *S. shirozui*); (II) odd elytral interstriae with interrupted pilose pustules, strial punctures very large, distance between punctures about 0.3 times a puncture diameter (vs. continuous pilose pustules, strial punctures large, distance between punctures subequal to puncture diameter in *S. shirozui*).

## 4. Discussion

The four new species of the tribe Stromboscerini described in this study highlight the diversity of this group in southwestern China. Although *D. tengchongensis* sp. nov. and *T. liangi* sp. nov. are each known from only one specimen, both possess diagnostic characters that reliably distinguish them from other morphologically similar species. Of particular interest is *T. biunciatus* sp. nov., which exhibits a unique combination of characters unknown in other members of the genus: male protibiae and mesotibiae each bear two apical unci and ventrite 5 is convex apically rather than emarginate. These distinctive features may indicate a unique evolutionary lineage. For instance, the presence of two apical unci on both the protibiae and mesotibiae represents a notable deviation from the typical single uncus in *Tasactes*. This character could reflect an adaptation to a specific microhabitat or movement behavior that reduced gene flow with congeners. Furthermore, the convex ventrite 5 is a state not seen in any species in the genus. The stable combination of these two uncommon characters is unlikely to be coincidental and more probably represents a suite of characters that evolved in an isolated population. Since the general morphology of this species conforms to the diagnosis of *Tasactes*, we place it in this genus. The absence of abdominal structures in the specimen prevented examination of the genitalia and additional collecting is needed to clarify phylogenetic position of *T. biunciatus* sp. nov.

Both this study and prior work [22] confirm that in the genus *Tasactes*, female sternite 8 serves as a reliable diagnostic character for species identification, providing complementary information to male genitalia. In contrast, the ovipositor and spermatheca do not serve as stable interspecific characters. These findings emphasize that comprehensive examination and documentation of internal genital structures in both sexes are essential for constructing a stable classification of Stromboscerini and related weevil groups.

Regarding the genus *Dexipeus*, we compared the syntype images of *D. costatus* and *D. fumosus* from Grebennikov [7,9] with the new species *D. tengchongensis*. This comparison confirms that “eyes distinctly narrowed ventrally” is a stable and notable synapomorphy for the genus. Consequently, we propose elevating this character to a key diagnostic feature of *Dexipeus*. The taxonomic status of *D. krasilnikovi* requires confirmation through direct examination of its type specimen due to the limited clarity of the images provided by Legalov [16].

In summary, the new taxa reported here enrich the known diversity of Stromboscerini and highlight several previously overlooked morphological characters. The complex topography and diverse habitats of the mountainous regions in southwestern China, particularly Mêdog County and the adjacent Gaoligong Mountains at the southeastern edge of the Himalayas, suggest a potentially rich yet undiscovered Stromboscerini fauna. Current study limitations include scarce material for some species and a lack of molecular phylogenetic data for critical taxa. Future research should combine targeted field surveys to obtain larger series (especially for species described from single specimens) with molecular systematics using standard markers to build a comprehensive phylogenetic framework. This integrated approach will test and refine the morphology-based classification, while detailed comparative morphology, particularly of male and female genitalia, will provide a robust evidence base for ultimately resolving the phylogeny and biogeographic history of the tribe Stromboscerini.

## Data Availability

The original contributions presented in this study are included in the article. Further inquiries can be directed to the corresponding author.
